# Total Distance Walked After Hip Surgery Identifies Older Patients With Sarcopenia

**DOI:** 10.1111/nhs.70169

**Published:** 2025-06-22

**Authors:** Pablo A. Marrero‐Morales, Enrique González‐Dávila, Eva M. Gallego‐González, Pablo Marrero‐Herrera, María Fernanda Hernández‐Gutiérrez

**Affiliations:** ^1^ Servicio de Rehabilitación Complejo Hospitalario Universitario de Canarias La Laguna Spain; ^2^ Departamento de Medicina Física y Farmacología Universidad de la Laguna La Laguna Spain; ^3^ Departamento de Matemáticas, Estadística e Investigación Operativa. IMAULL Universidad de La Laguna La Laguna Spain; ^4^ Servicio de Traumatología Complejo Hospitalario Universitario de Canarias La Laguna Spain; ^5^ Universidad Europea de Canarias, Sección de Enfemería La Orotava Spain

**Keywords:** functional physical performance, functional status, geriatrics, hip fracture, sarcopenia

## Abstract

This study aims to determine the optimal cut‐off point for total walking distance on the day of hospital discharge to identify older adults at high risk of sarcopenia following hip fracture surgery. A prospective observational study included 135 patients aged > 65 years who underwent hip fracture surgery. Logistic regression models were used to predict probable or confirmed sarcopenia based on hospital stay data. Cut‐off points were determined using the Youden index, with internal validation through 10‐fold cross‐validation. The mean age of participants was 81.6 years (SD 7.6), and 72% were women. Sarcopenia was suspected in 33% of patients, with half meeting criteria for confirmed sarcopenia. Walking distance on discharge decreased significantly with increasing sarcopenia severity (*p* < 0.001). Thresholds of ≤ 8.8 m for women and ≤ 11.3 m for men identified sarcopenia, with positive predictive values of 90% and 62.5%, and negative predictive values of 73.7% and 96.7%, respectively. Alternative models were developed for cases lacking walking distance data. Walking distance at discharge is a simple, practical, and effective marker for identifying sarcopenia in older adults after hip fracture surgery.

**Summary**

- Sarcopenia is a geriatric syndrome that affects the musculoskeletal system and is associated with an increase in falls and hip fractures.
- The determination of the degree of sarcopenia requires specific and expensive tests often difficult to perform in daily clinic.
- This paper provides optimal cut‐off points for the total distance traveled on the day of hospital discharge after a hip fracture, which identify patients with a high probability of having sarcopenia.

## 1 Introduction

Countries around the world are undergoing a demographic transition, characterized by an increasing number and proportion of individuals aged 65 and older compared to children under the age of five (Ritchie and Roser 2024). This shift has led to high health dependency rates among the older, non‐working population, particularly in Europe, North America, and East Asia (Ritchie and Roser 2024; Harasty and Ostermeier 2020).

The increase in life expectancy has led to a growing number of older individuals and a concurrent rise in age‐related diseases (Lunenfeld and Stratton 2013). Conditions such as lung, breast, and colon cancer, cardiovascular diseases, metabolic syndrome, reduced skeletal bone mass, and geriatric syndromes significantly correlate to accidental falls (Viganò et al. 2023).

Hip fractures in older adults worldwide have been rising over the last decades, especially in Europe, Asia, and the United States (Haleem et al. 2023; Amusan et al. 2019) and are expected to reach up to 21.3 million worldwide by 2050 (Viganò et al. 2023). This increment represents an enormous challenge for universal public health systems due to the significant economic impact and high morbidity and mortality (Feng et al. 2024).

Many of these patients recover their previous functionality completely or partially after surgery. The parameters used to assess the functional recovery of these older patients who have undergone hip fracture surgery are the ability to perform basic and instrumental activities of daily living, as well as their walking ability (Kujala et al. 2023; Ahmed et al. 2023).

Of these parameters, walking ability is the most critical, given its significant impact on the patient's daily life and its relevance in routine clinical practice. This ability is closely linked to the distance that the patient is able to walk with technical aids after the intervention, in physiotherapy treatment during hospital stay (Kujala et al. 2023; Fisher et al. 2023).

Several factors influence the functional recovery of these patients and may explain the differences observed in their postoperative outcomes. These include the type of osteosynthesis material used (López‐Hualda et al. 2023), the nature of the fracture (Terhune and Williams 2022), pain levels (Goto et al. 2020), cognitive function (Uzoigwe et al. 2020; Liu et al. 2022), and, notably, the patient's muscle strength, skeletal muscle mass, and physical performance. A decline in any of these three last factors, which are interrelated and play a crucial role in mobility, balance, and the ability to perform daily activities, is associated with sarcopenia, a recognized health condition linked to poorer recovery outcomes (Chang et al. 2023; Huang et al. 2021).

Sarcopenia is a geriatric syndrome that affects the musculoskeletal system, impairing walking ability (Morley et al. 2011), as well as the capacity to perform basic and instrumental activities of daily living. It also leads to a general decline in quality of life, increasing morbidity and mortality among those affected (Bobovec et al. 2023). The decrease in muscle mass and strength in sarcopenia leads to alterations in neuromuscular function and muscle fiber quality, resulting in a decreased ability to generate force and, consequently, a reduction in walking distance. This is particularly relevant in patients undergoing hip fracture surgery, where the pre‐existing loss of muscle mass can be exacerbated by surgical stress and the period of postoperative immobility (Morley et al. 2011; Bobovec et al. 2023).

In 2018, the European Working Group on Sarcopenia in Older People (EWGSOP2) (Cruz‐Jentoft et al. 2019) established sarcopenia assessment tests and cut‐off points using a sequential algorithm. The initial phase involves screening with the Sarc‐F questionnaire, which evaluates the clinical risk of sarcopenia (Drey et al. 2020). If the Sarc‐F result is positive, sarcopenia classification is performed to confirm the final diagnosis (Kim and Won 2019). However, current confirmatory assessments—such as DXA (dual‐energy X‐ray absorptiometry), bioimpedance analysis, or gait speed tests—are often underutilized in routine clinical practice due to their cost, limited accessibility, technical complexity, and time requirements (Borda et al. 2024; Ackermans et al. 2022). These barriers limit the widespread identification and management of sarcopenia, particularly in high‐pressure hospital settings.

Therefore, there is a clear need for simple, low‐cost, and feasible tools that can help identify patients with probable sarcopenia early during hospitalization.

The main objective of this study is to determine the optimal cut‐off point for the total distance walked on the day of hospital discharge, after surgical treatment of hip fracture, in order to identify older patients with a high probability of having sarcopenia.

## 2 Material and Methods

A prospective observational study was conducted at a tertiary‐level hospital center. Patients over 65 years of age admitted to the orthopedic surgery and traumatology ward for surgery with a diagnosis of hip fracture between November 2019 and April 2020 who were requested physiotherapy treatment were included in the sample. Patients scheduled for hip surgery due to conditions such as osteoarthritis are not included.

Patients who were unable to walk before surgery, those who were not allowed to bear weight on their affected lower limb, patients with stroke, those with upper limb fractures that prevented them from using a walker or cane, and pathological tumorous hip fracture were excluded.

The research was conducted in accordance with the Declaration of Helsinki and received the approval of the Biomedical Research Ethics Committee of the hospital (CHUC_2019_80). Voluntary exclusion was used for informed consent of all participants.

### 2.1 Participant Characteristics

**FIGURE 1:**
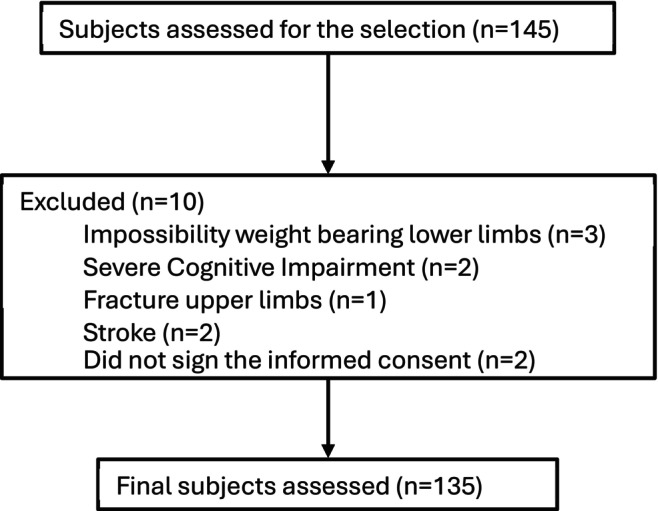
Flow chart.

The sample size was calculated to detect a minimum difference of 1 m in walking distance after hip fracture surgery between patients with and without sarcopenia, assuming a standard deviation of 2 m, a 95% confidence level, 80% statistical power and an expected 10% loss to follow‐up or failure to meet inclusion criteria. Based on these parameters, a minimum of 144 participants was estimated to be required. This figure includes the 10% increase to account for potential losses, meaning the core sample size without adjustment was 131 participants. Initially, 145 participants were evaluated, of which 10 were excluded for not meeting the inclusion criteria. The final analytical sample consisted of 135 participants, which exceeds the minimum required sample size (*n* = 131) and is therefore considered sufficient for the planned statistical power. Data were collected by the same researcher (first author) (Figure 1).

### 2.2 Procedure

The study collected information in two periods, at hospital admission and at discharge. The data were collected from the patient's own medical history during their hospital stay. The information obtained included age, sex, fracture type, date of admission and surgery, comorbidities according to the Charlson index (Charlson et al. 1987) and presence of delirium according to the Confusion Assessment Method (CAM) criteria (Inouye et al. 1990).

Additionally, the patient's baseline status (prior to the hip fracture) was recorded, including assessments of basic activities of daily living with the Katz Index (Katz 1963), which evaluates independence in six essential self‐care functions (bathing, dressing, toileting, transferring, continence, and feeding); instrumental activities using the Lawton & Brody scale (Lawton et al. 1969) (Lawton), which assesses more complex daily tasks such as using the telephone, managing finances, and medication; and assistance required for walking using the Functional Ambulation Classification (Viosca et al. 2005) (FAC), a simple clinical scale that classifies ambulation ability into six levels (from 0 = non‐functional ambulation to 5 = independent ambulation on all surfaces). Additionally, at admission, cognitive function was assessed using the Pfeiffer questionnaire (Marrero‐Morales et al. 2023), a brief screening tool for detecting cognitive impairment based on 10 questions addressing orientation, memory, and calculation.

Sarcopenia assessment, following the EWGSOP2 guidelines, began with the completion of the Sarc‐F questionnaire (Cruz‐Jentoft et al. 2019) a five‐item screening tool that assesses strength, walking ability, ability to rise from a chair, stair climbing, and history of falls. A score ≥ 4 indicates a high risk of sarcopenia. Patients with a positive screening (Sarc‐F ≥ 4) who presented low muscle strength assessed by isometric dynamometry (Mathiowetz et al. 1984; Lauretani et al. 2003) of the dominant hand (grip strength) with a validated “JAMAR” dynamometer were classified as having probable sarcopenia. If patients additionally presented a decrease in muscle mass, evaluated in this work by bioelectrical impedance (Lukaski 1991) with a MALTRON BF‐905 analyzer, they were classified as having confirmed sarcopenia. Within this last group, those patients who also presented a low physical performance assessed by the Short Physical Performance Battery (SPPB) (Pavasini et al. 2016) that evaluates balance, walking, strength, and resistance, were identified as having severe sarcopenia. In this study, sarcopenia is identified when any of the three previously mentioned criteria are fulfilled.

From the first day after surgery through the day of hospital discharge, all patients received standard physiotherapy treatment for 45 min daily. These sessions included muscle strengthening exercises and balance training (squats, knee bends, static steps and walking with a walker). The total distance covered was calculated by summing the four walking trials performed with a walker during the physiotherapy sessions, under therapist supervision. No physical assistance was provided during ambulation, except for minimal support to ensure balance. The distance for each trial was measured using a homologated extendable tape measure. In addition, personalized rehabilitation techniques were also added based on the needs and deficiencies of each patient to improve the gait and stability of each individual.

### 2.3 Statistical Analysis

The information on continuous variables is presented showing mean ± standard deviation (s.d.) when they follow a normal distribution, evaluated with the Kolmogorov–Smirnov test, and otherwise, with median and interquartile range. For categorical variables, absolute frequencies and percentages were used.

The comparison of the results of the different variables collected with respect to the degree of sarcopenia was carried out using ANOVA or the Kruskal–Wallis test in the case of parametric or non‐parametric continuous variables, respectively, and with the Chi‐square test for categorical variables. The relationship between continuous variables was evaluated with the Pearson or Spearman correlation coefficient. Logistic regression was applied with the forward Wald variable selection method to obtain the models that explain having sarcopenia or confirmed sarcopenia. The determination of the optimal cut‐off points for the total distance walked, FAC, as well as the estimated probability according to the models obtained, was carried out using the Youden index, that is, the balance point between sensitivity (SENS) and specificity (SPEC) of the test. The positive predictive value (PPV) and negative predictive value (NPV) under the probabilities of sarcopenia and confirmed sarcopenia of the sample are included, as well as the 95% confidence intervals (95% CI). Receiver operating characteristic (ROC) curves and the area under them (AUC) are also provided and compared.

Internal validation of the models was carried out by applying logistic regression using 10‐fold cross validation. True positive rates (equivalent to SENS and SPEC depending on response category), false positive rates (1‐SPEC and 1‐SENS), precision (PPV and NPV), F‐Measure index (2 × True positive rate × precision/(true positive rate + precision)) and Cohen's Kappa agreement coefficient are shown.

Statistical analyses were performed with SPSS v29 (IBM SPSS, Armonk, NY) and MedCalc v19.2.6 (MedCalc, Ostend, Belgium). Model validation was performed using the Weka v3.8 program (Machine learning, University of Waikato, NZ). Results were considered statistically significant if *p* < 0.05.

## 3 Results

The characteristics of the patients evaluated depending on the degree of sarcopenia are shown in Table 1. Of the 135 patients with hip fracture analyzed, 90 (67%) did not present sarcopenia, compared to 45 (33%) who did, 21 of them classified as having probable sarcopenia and 24 as having confirmed sarcopenia (16 of them severe). The mean age of the patients is 81.6 ± 7.6 years, although it increases significantly depending on the degree of sarcopenia (*p* = 0.002), with 75% of patients in the confirmed sarcopenia group being 85 years or older. 71.9% of the total were women, although these percentages also increased significantly with the degree of sarcopenia (*p* = 0.019), going from 64.4% in the group without sarcopenia to reaching 91.7% in the group with confirmed sarcopenia. Comorbidities, measured through the Charlson index, also show this same behavior (*p* = 0.002). Activities of daily living (Katz), instrumental activities (Lawton) and ambulation functionality (FAC), all show a significant worsening the higher the degree of sarcopenia (all *p* ≤ 0.004), as well as a worse cognitive status (*p* = 0.047).

**TABLE 1:** Patient characteristics depending on the degree of sarcopenia.

	Degree of sarcopenia			*p*	Total (*N* = 135)
	Non‐Sarcopenia (*N* = 90)	Probable (*N* = 21)	Confirmed (*N* = 24)		
Age (years)	80.3 ± 7.6	82.0 ± 5.9	86.3 ± 7.2	0.002	81.6 ± 7.6
Age, *n* (%)				0.004	
< 75	19 (21.1)	3 (14.3)	3 (12.5)		25 (18.4)
[75, 85)	42 (46.7)	10 (47.6)	3 (12.5)		55 (40.8)
≥ 85	29 (32.2)	8 (38.1)	18 (75.0)		55 (40.8)
Female, *n* (%)	58 (64.4)	17 (81.0)	22 (91.7)	0.019	97 (71.9)
Body mass index (kg/m^2^)	26.9 ± 4.3	28.7 ± 4.3	24.7 ± 4.2	0.009	26.8 ± 4.4
Charlson index	4.5 ± 1.3	5.0 ± 1.5	5.7 ± 1.8	0.002	4.8 ± 1.5
Delirium, *n* (%)	5 (5.6)	2 (9.5)	3 (12.5)	0.474	10 (7.4)
Type of fracture				0.044	
Intracapsular	40 (44.4)	7 (33.3)	10 (41.7)		57 (42.2)
Subtrochanteric	8 (8.9)	7 (33.3)	2 (8.3)		17 (12.6)
Pertrochanteric	42 (46.7)	7 (33.3)	12 (50.0)		61 (45.2)
Pain on discharge day				0.023	
No pain/mild	47 (52.2)	5 (25.0)	7 (29.2)		59 (44.0)
Moderate/very severe	43 (47.8)	15 (75.0)	17 (70.8)		75 (56.0)
Functional tests					
Katz					
Average ± s.d.	4.7 ± 0.8	4.0 ± 1.2	4.2 ± 1.6	0.004	4.5 ± 1.1
Katz, *n* (%)				0.024	
Severe disability (0–2)	3 (3.3)	3 (14.3)	3 (12.5)		9 (6.7)
Moderate disability (3, 4)	15 (16.7)	8 (38.1)	6 (25.0)		29 (21.4)
Mild or no disability (5, 6)	72 (80.0)	10 (47.6)	15 (62.5)		97 (71.9)
Lawton					
Average ± s.d.	5.5 ± 2.7	4.1 ± 2.3	2.9 ± 2.3	< 0.001	4.8 ± 2.7
Median (P_25_; P_75_)	6.2 (3.2; 8.0)	4 (1.8; 6.0)	2.4 (1; 4.9)	< 0.001	5 (2: 8)
Lawton, *n* (%)				0.002	
Severe dependence (0–2)	16 (17.8)	7 (33.3)	12 (50.0)		35 (25.9)
Mild dependence (3–5)	24 (26.7)	8 (38.1)	8 (33.3)		40 (29.6)
Independence (6–8)	50 (55.5)	6 (28.6)	4 (16.7)		60 (44.4)
FAC					
Average ± s.d.	4.4 ± 0.7	4.0 ± 0.6	3.7 ± 1.0	< 0.001	4.2 ± 0.8
FAC, *n* (%)				0.001	
Walking with aid (0–1)	1 (1.1)	—	1 (4.2)		2 (1.5)
Supervised walking (2, 3)	4 (4.4)	4 (19.0)	8 (33.3)		16 (11.9)
Independent walking (4, 5)	85 (94.5)	17 (81.0)	15 (62.5)		117 (86.7)
Cognitive impairment					
Pfeiffer					
Average ± s.d.	3.5 ± 2.5	4.2 ± 2.4	4.9 ± 2.5	0.047	3.9 ± 2.5
Median (P_25_; P_75_)	3 (1; 5)	4 (2; 6)	5 (3; 7)	0.048	4 (2; 6)
Pfeiffer, *n* (%)				0.305	
No impairment (0–2)	34 (37.8)	6 (28.6)	5 (20.8)		45 (33.3)
Mild impairment (3, 4)	23 (25.5)	5 (23.7)	5 (20.8)		33 (24.4)
Moderate impairment (5–7)	26 (28.9)	9 (42.9)	9 (37.5)		44 (32.6)
Severe impairment (8–10)	7 (7.8)	1 (4.8)	5 (20.8)		13 (9.6)
Total distance walked (m)					
Average ± s.d.	28.6 ± 16.7	8.7 ± 10.1	4.5 ± 2.9	< 0.001	21.7 ± 17.6
Median (P_25_; P_75_)	23 (15; 39.8)	4.5 (2.4; 11.8)	4.7 (2.5; 5.5)	< 0.001	17.2 (6.3; 33.6)

Abbreviations: FAC, functional ambulation classification; s.d., standard deviation.

The test of the total distance walked on the discharge day also shows a significant decrease the worse the degree of sarcopenia (*p* < 0.001), going from an average walk of 28.6 m in the non‐sarcopenia group to 4.5 m in the confirmed sarcopenia group. We did not find significant differences based on the three types of fracture and osteosynthesis considered (*p* = 0.252) after controlling for sex and sarcopenia classification.

**FIGURE 2:**
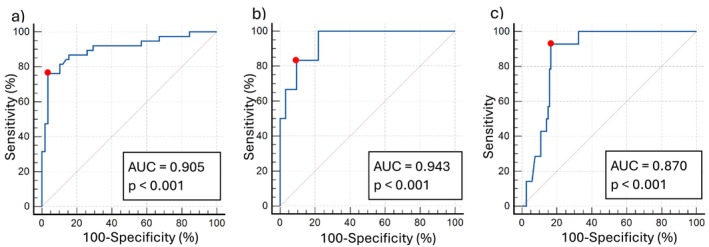
ROC curves for sarcopenia in (a) women and (b) men and for (c) confirmed sarcopenia (the red dot shows the cut‐off point according to the Youden index and AUC is the area under curve).

The resulting discrimination models for the degrees of sarcopenia are shown in Table 2. For both men and women, when discriminating between non‐sarcopenia and sarcopenia, as well as for the identification of confirmed sarcopenia, the only variable that enters the models, once the forward Wald variable selection method is applied, is the total distance walked on discharge day. Figure 2 shows the ROC curves for these three models.

**TABLE 2:** Logistic regression models for the probability of having (a) sarcopenia, depending of sex and (b) confirmed sarcopenia.

Models to	Coefficient	s.e.	Wald	*p*	Odds ratio (95% CI)	Cut‐off points	
						Distance (m)	$\hat{p}$ (%)
(a) Sarcopenia							
Female						≤ 8.8	> 51.61
Constant	1.979	0.513	14.880	< 0.001	7.238		
Total distance walked (m)	−0.170	0.038	20.025	< 0.001	0.844 (0.783; 0.909)		
Male						≤ 11.3	> 46.25
Constant	1.723	1.147	2.258	0.133	5.601		
Total distance walked (m)	−0.240	0.106	5.155	0.023	0.786 (0.639; 0.968)		
(b) Confirmed sarcopenia							
						≤ 6.0	> 19.84
Constant	0.021	0.485	0.002	0.965	1.022		
Total distance walked (m)	−0.222	0.068	10.486	0.001	0.801 (0.701; 0.916)		

Abbreviations: CI, confidence interval; $\hat{p}$, estimated probability of sarcopenia or confirmed sarcopenia according to the models ($\hat{p}=1/\left(1+\exp(-\mu \left(x\right)\right)$ with $\mu \left(x\right)$ the linear predictor); s.e., standard error.

In the case of identifying the presence of sarcopenia, for both men and women, the area under the ROC curve is greater than 0.9 (women 0.905, 95% CI (0.828; 0.955) and men 0.943, 95% CI (0.816; 0.992), both with *p* < 0.001). In particular, those women who walk 8.8 m or less (SENS = 76.3%, 95% CI (59.8; 88.6); SPEC = 96.6%, 95% CI (88.1; 99.6): PPV = 90.0%, 95% CI (68.9; 97.3) and NPV = 73.7%, 95% CI (67.4; 79.2)) and men who walk 11.3 m or less (SENS = 83.3%, 95% CI (35.9; 99.6); SPEC = 90.6%, 95% CI (75.0; 98.0); PPV = 62.5%, 95% CI (34.9; 83.8) and NPV = 96.7%, 95% CI (82.8; 99.4)) will be identified as having sarcopenia.

**FIGURE 3:**
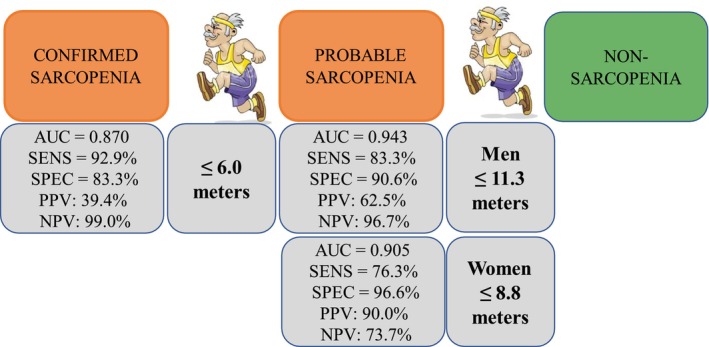
Cut off points for the classification of degrees of sarcopenia.

The model for the identification of confirmed sarcopenia presents an area under the ROC curve of 0.870, 95% CI (0.801; 0.922) with *p* < 0.001. Those patients who walk 6.0 m or less will be identified with confirmed sarcopenia (SENS = 92.9%, 95% CI (66.1; 99.8); SPEC = 83.3%, 95% CI (75.4; 89.5); PPV = 39.4%, 95% CI (29.8; 49.9) and NPV = 99.0%, 95% CI (93.8; 99.8)). Figure 3 summarizes the information on the cut‐off points for the classification of degrees of sarcopenia.

If the distance walked test on the discharge day was not available, Table S1 shows the resulting models. To identify sarcopenia, the variables introduced into the model would be sex, the Charlson index, and the Functional Ambulation Classification (FAC) test score. The area under the ROC curve is 0.705, 95% CI (0.706, 0.851) with *p* < 0.001. In this case, a patient will be identified with sarcopenia when the estimated probability of sarcopenia according to the model exceeds 24.32% (SENS = 80.0%, 95% CI (65.4; 90.4), SPEC = 64.4%, 95% CI (53.7; 74.3), PPV = 52.9%, 95% CI (45.1; 60.6), and NPV = 86.6%, 95% CI (77.9; 92.2)). Figure S1a shows the comparison of the ROC curves using this model and the one that includes only the total distance walked, with that of the total distance walked being significantly higher (*p* = 0.009).

The resulting model to identify patients with confirmed sarcopenia when the total distance walked is not available includes only the Functional Ambulation Classification (FAC) test score. The area under the ROC curve is 0.692, 95% CI (0.6076; 0.768) with *p* = 0.009. A patient will be classified as having confirmed sarcopenia when the FAC score is 3 or less (supervised or assisted walking), or equivalently, the estimated probability according to the model exceeds 10.48% (SENS = 57.1%, 95% CI (28.9; 82.3), SPEC = 75.2%, 95% CI (66.5; 82.6), PPV = 21.1%, 95% CI (13.3; 31.6), and NPV = 93.8%, 95% CI (89.1; 96.6)). Figure S1b shows the comparison of the ROC curves of this model versus that of the model that uses only the distance walked (*p* = 0.022).

The internal validation of the proposed models is shown in Table S2. The models for predicting sarcopenia in men and women that use walked distance present Kappa concordance coefficients that exceed the value of 0.6, the areas under the ROC curve are close to 0.9, the average true positive rates exceed 0.83 (always better in non‐sarcopenia) and average false positive rates below 0.3 (worse in non‐sarcopenia and generally worse in men than in women). For the rest of the models, that is, the model that uses sex, the Charlson index and FAC for sarcopenia, as well as the distance and FAC score for confirmed sarcopenia, the Kappa concordance indices are lower, with values below 0.33, with true positive rates on non‐sarcopenia or non‐confirmed relatively high, exceeding 0.8, but somewhat lower on sarcopenia or confirmed sacopenia, and false positive rates exceeding 0.4.

## 4 Discussion

The determination of the degree of sarcopenia requires specific and expensive tests often difficult to perform in daily clinic. The strength of this study is that the measurement of the total distance walked is a common test in the clinical protocols of physiotherapy and traumatology of patients with hip fracture, and the results included show that it is useful for discriminating the existence of sarcopenia. In the literature reviewed, there are few previous studies that address the total distance walked as a predictor of the degree of sarcopenia, which gives special relevance to the contribution of our study.

The sarcopenia screening test using the total distance walked on the day of discharge, in the case of women, can be classified as good for confirming the disease. Specifically, if you walk less than 8.8 m, the probability of having sarcopenia is 90% (PPV = 90%). However, its utility for ruling out the disease is somewhat lower, as walking more than 8.8 m corresponds to a 26.3% probability of having sarcopenia (NPV = 73.7%). In men, the behavior is slightly different: the test is good for ruling out sarcopenia, with a probability of not having the condition at 96.7% if you walk more than 11.3 m (NPV = 96.7%). Conversely, its utility for confirmation is somewhat lower, as walking less than 11.3 m corresponds to a 62.5% probability of having sarcopenia (PPV = 62.5%). The differences observed between men and women in the relationship between distance walked and the likelihood of sarcopenia could be explained by several physiological and biological factors. Women generally have a higher proportion of body fat, which could influence their ability to walk a given distance, compared to men, who tend to have more muscle mass. Furthermore, previous studies have shown that the prevalence and progression of sarcopenia may differ between genders, which could explain the different thresholds established in our study (Lee et al. 2008). Furthermore, social and cultural factors that affect physical activity levels may also play a role in the observed differences. In men, greater physical activity or greater overall muscle mass could be associated with greater endurance and the ability to walk longer distances (Fragala et al. 2012).

The total walking distance test is useful to rule out confirmed sarcopenia. Those patients who walk more than 6.0 m have a 99% probability of not presenting it (NPV = 99.0%). Patients who walk less than 6.0 m have only a 39.4% probability of confirmed sarcopenia (PPV = 39.4%). When the total walking distance is unavailable, the proposed models for both sarcopenia and confirmed sarcopenia are primarily effective for ruling out the disease. The study by Nanri et al. (2024) concludes that positive results in the risk of sarcopenia were associated with a delay in independent postoperative walking after hip surgery, regardless of sex, age, BMI, and comorbidities. These results are consistent with this work. The ability to walk and post‐surgical recovery from a hip fracture are directly related to the stimulation of bone regeneration. Regular movement can help improve blood circulation, which promotes bone nutrition and bone repair after a fracture. Furthermore, the mechanical load imposed on the bone during walking could be an important factor in bone remodeling (Bader et al. 2011). Loss of muscle mass and strength can negatively affect walking ability. Decreased muscle strength can hamper mobility and, in turn, delay recovery from hip fracture. Furthermore, sarcopenia has been associated with increased bone fragility, which could influence both the bone healing process and the patient's ability to perform postoperative rehabilitation (Beaudart et al. 2016). They often present with muscle weakness, which can increase the risk of falls, fractures, and postoperative complications (Kujala et al. 2023).

Duke et al. (Duke and Keating 2002) in a cohort study of patients admitted with hip fracture without sarcopenia, found that mobility measurements 2 days after surgery were the main predictors of independence in transfers and walking. Marrero‐Morales et al. (2023) also found that sarcopenia was one of the main factors explaining functional recovery and walking 1 year after surgery.

Gherardini et al. (2019) concluded that walking speed when walking four meters, measured after surgery for a hip fracture in older people without sarcopenia, is important for long‐term prognosis. This assessment method, although intended for patients without sarcopenia, could also be a method to be studied for the discrimination of patients with sarcopenia. Parker et al. (2010) indicate that hip intracapsular tends to offer a faster recovery compared to internal fixation of fractures (Pertrocanterea y subtrocantera) as it allows for a more complete restoration of joint mobility. However, our results for distance walked on the day of discharge show no significant differences or clear trends based on fracture type.

Among the limitations of the work is the small number of male patients, which is probably the reason why we cannot define a specific model within the confirmed sarcopenia group. Despite having carried out internal validation tests of the proposed models, it would be advisable to carry out an external validation of these models and cut‐off points using new data, both within the same geographical area and in different geographical areas. It should also be noted that there is no discrimination based on severe sarcopenia due to having a small sample. Additionally, the generalizability of these findings should be interpreted with caution, as factors such as cognitive impairment, differences in walking rehabilitation plans, and the severity of other conditions affecting mobility may influence the results. Other potential confounding factors that may interfere with walking ability include comorbidities, chronic illnesses, and medications associated with the older population.

## 5 Relevance for Clinical Practice

This study would help identify older patients at high risk of sarcopenia based on the total distance walked on the day of hospital discharge after surgical treatment for hip fracture. Early detection of sarcopenia would allow for the adaptation and personalization of intervention protocols in nursing, physiotherapy, and medicine. However, the generalizability of these findings should be interpreted with caution, as factors such as cognitive impairment, differences in walking rehabilitation plans, and the severity of other conditions affecting mobility may influence the results.

## 6 Conclusion and Implications

The determination of optimal cut‐off points on the total distance walked test on the day of hospital discharge, after surgical treatment of hip fracture, to identify older patients with a high probability of presenting sarcopenia is the greatest contribution of the present study. Those patients who walk a total distance less than or equal to 8.8 m (women, sensitivity: 76.9%; specificity: 98.3%) or 11.3 m (men, sensitivity: 87.5%, specificity: 96.7%), have a high probability of having sarcopenia. In addition, if this distance is less than 6.0 m, the probability of having confirmed sarcopenia increases significantly.

The determination of the degree of sarcopenia requires more specific and expensive tests to perform in clinical practice. In contrast, the total distance walked test is simple, inexpensive, and widely used in physiotherapy protocols. Our findings support its integration as a screening tool in standard rehabilitation pathways following hip fracture surgery, allowing early identification of patients at risk of sarcopenia and the implementation of targeted interventions to improve recovery outcomes.

## Author Contributions

**Pablo A. Marrero‐Morales:** data collection, study design, analysis, writing and verification. **Enrique González‐Dávila:** statistical analysis and data interpretation. **María Fernanda Hernández‐Gutiérrez:** study design and writing. **Eva M. Gallego‐González:** study design and writing. **Pablo Marrero‐Herrera:** study design and writing. All authors read, corrected, and approved the final version submitted.

## Ethics Statement

This study has received a favorable opinion by the Biomedical Research Ethics Committee of the University Hospital Complex of the Canary Islands (CHUC_2019_80).

## Conflicts of Interest

The authors declare no conflicts of interest.

## Supporting information


**Data S1.** Supporting Information.

**Figure S1.** Comparison of ROC curves for (a) sarcopenia of the models with only distance and the one that uses sex, the Charlson index and Functional Ambulation Classification (FAC) and, for (b) confirmed sarcopenia of the models with only distance and the one that uses only FAC.

**Table S1.** Logistic regression models for the probability of having (a) sarcopenia and (b) confirmed sarcopenia when total distance walked is not available.
**Table S2.** Internal cross‐validation 10‐fold for the models described in Table 2 and Table S1.

## Data Availability

The data that support the findings of this study are available on request from the corresponding author. The data are not publicly available due to privacy or ethical restrictions.
