# Supplementary material for: Total Distance Walked After Hip Surgery Identifies Older Patients With Sarcopenia
Source: Nurs Health Sci. 2025 Jun 22;27(2):e70169. doi: 10.1111/nhs.70169 (PMC12183496; doi:10.1111/nhs.70169)
Supplement: Supplementary file 2 — Figure S1. Comparison of ROC curves for (a) sarcopenia of the models with only distance and the one that uses sex, the Charlson index and Functional Ambulation Classification (FAC) and, for (b) confirmed sarcopenia of the models with only distance and the one that uses only FAC. [file NHS-27-e70169-s002.docx]

**Figure S1**


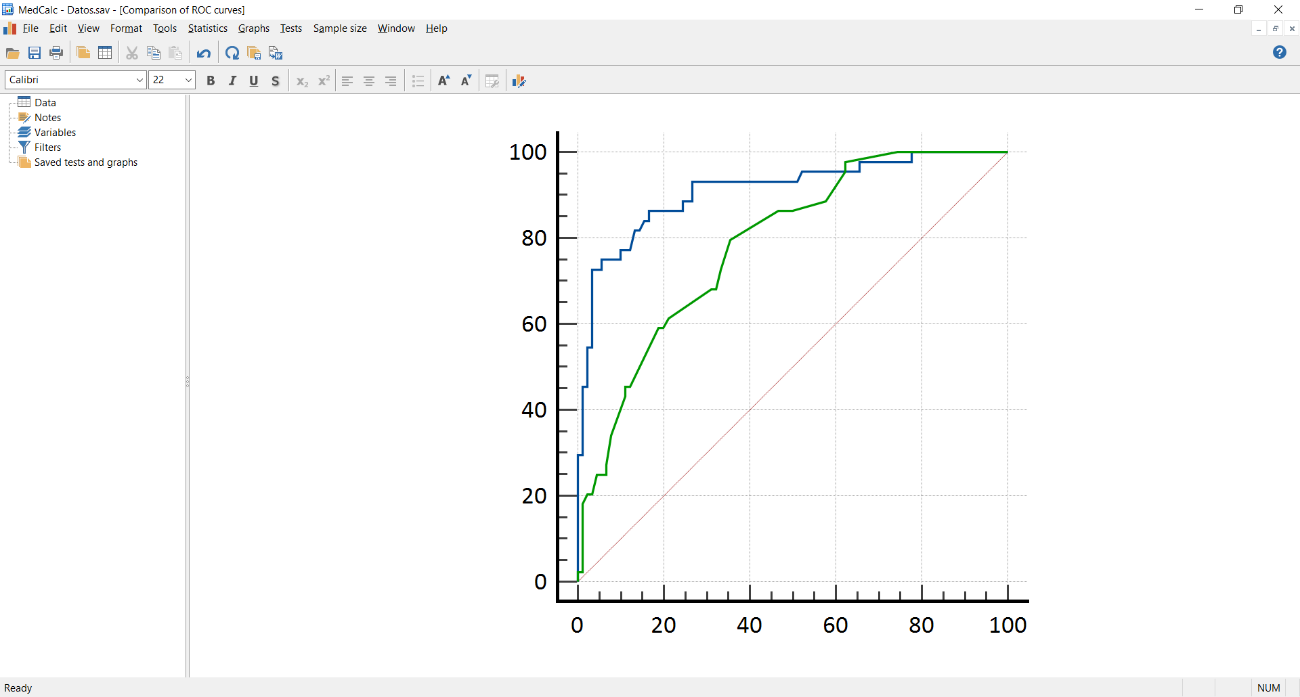


Distance

Sex, Charlson and FAC

Sensitivity (%)

100-Specificity (%)

a)


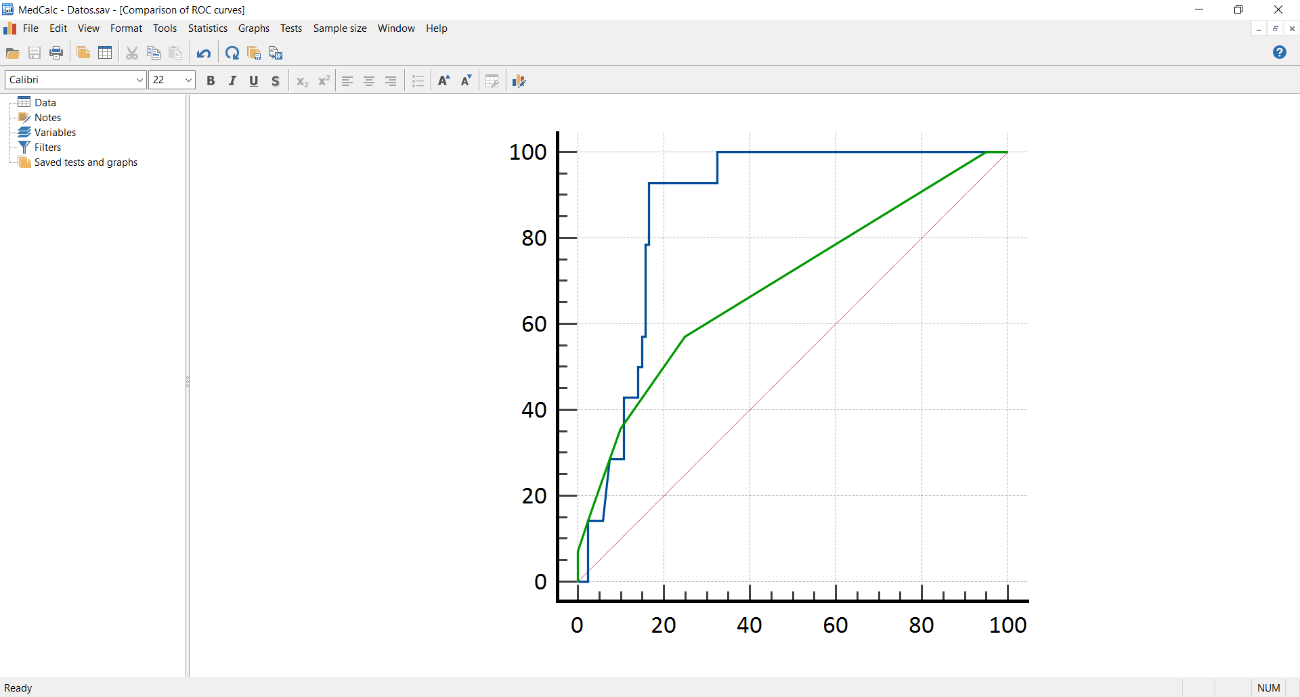


Distance

FAC

Sensitivity (%)

100-Specificity (%)

b)
