# Supplementary material for: Total Distance Walked After Hip Surgery Identifies Older Patients With Sarcopenia
Source: Nurs Health Sci. 2025 Jun 22;27(2):e70169. doi: 10.1111/nhs.70169 (PMC12183496; doi:10.1111/nhs.70169)
Supplement: Supplementary file 3 — Table S1. Logistic regression models for the probability of having (a) sarcopenia and (b) confirmed sarcopenia when total distance walked is not available. Table S2. Internal cross‐validation 10‐fold for the models described in Table 2 and Table S1. [file NHS-27-e70169-s003.docx]

**Table S1**

| Models to | Coefficient | s.e. | Wald | p-value | Odds ratio (CI_95%_) | Cut-off points | |
| --- | --- | --- | --- | --- | --- | --- | --- |
| 1. Sarcopenia |  |  |  |  |  | $\hat{p}$ (%) | |
|  |  |  |  |  |  | > 24.32 | |
| Constant | 0.474 | 1.621 | 0.085 | 0.770 | 1.606 |  | |
| Female | 1.299 | 0.539 | 5.804 | 0.016 | 3.666 (1.274; 10.549) |  | |
| Charlson index | 0.346 | 0.146 | 5.649 | 0.017 | 1.414 (1.063; 1.881) |  | |
| FAC | -0.928 | 0.293 | 10.058 | 0.002 | 0.395 (0.223; 0.702) |  | |
| 1. Confirmed sarcopenia | |  |  |  |  | FAC | $\hat{p}$ (%) |
|  |  |  |  |  |  | ≤ 3 | > 10.48 |
| Constant | 1.057 | 1.198 | 0.779 | 0.378 | 2.878 |  |  |
| FAC | -0.801 | 0.304 | 6.933 | 0.008 | 0.449 (0.247; 0.815) |  |  |

s.e.: standard error; CI: Confidence Interval; FAC: Functional Ambulation Classification; $\hat{p}$: estimated probability of sarcopenia or confirmed sarcopenia according to the models ($\hat{p}=1/\left( 1+exp(-\mu(x) \right)$ with $\mu(x)$ the linear predictor).

**Table S2**

| Models to | TP Rate | FP Rate | Precision | F-Measure | AUC | Kappa |
| --- | --- | --- | --- | --- | --- | --- |
| 1. Sarcopenia |  |  |  |  |  |  |
| Only distance |  |  |  |  |  |  |
| Female |  |  |  |  | 0.882 | 0.657 |
| Non-sarcopenia | 0.862 | 0.205 | 0.862 | 0.862 |  |  |
| Sarcopenia | 0.795 | 0.138 | 0.795 | 0.795 |  |  |
| Weighted average | 0.835 | 0.178 | 0.835 | 0.835 |  |  |
| Male |  |  |  |  | 0.896 | 0.604 |
| Non-sarcopenia | 0.938 | 0.333 | 0.938 | 0.938 |  |  |
| Sarcopenia | 0.667 | 0.063 | 0.667 | 0.667 |  |  |
| Weighted average | 0.895 | 0.291 | 0.895 | 0.895 |  |  |
| Sex, Charlson and FAC |  |  |  |  | 0.765 | 0.327 |
| Non-sarcopenia | 0.878 | 0.578 | 0.752 | 0.810 |  |  |
| Sarcopenia | 0.422 | 0.122 | 0.633 | 0.507 |  |  |
| Weighted average | 0.726 | 0.426 | 0.713 | 0.709 |  |  |
| 1. Confirmed sarcopenia | |  |  |  |  |  |
| Only distance |  |  |  |  | 0.732 | 0.302 |
| Non-confirmed | 0.809 | 0.458 | 0.890 | 0.848 |  |  |
| Confirmed | 0.542 | 0.191 | 0.382 | 0.448 |  |  |
| Weighted average | 0.761 | 0.410 | 0.799 | 0.776 |  |  |
| FAC |  |  |  |  | 0.667 | 0.189 |
| Non-confirmed | 0.856 | 0.667 | 0.856 | 0.856 |  |  |
| Confirmed | 0.333 | 0.144 | 0.333 | 0.333 |  |  |
| Weighted average | 0.763 | 0.574 | 0.763 | 0.763 |  |  |

TP: True positive; FP: False positive; AUC: Area under curve; FAC: Functional Ambulation Classification
